# New Player in the Field of Glioblastoma Therapy: EGFRvIII-Specific Gol1 Aptamer Shows a Great Therapeutic Potential

**DOI:** 10.3390/pharmaceutics18030299

**Published:** 2026-02-27

**Authors:** Fatima Dzarieva, Svetlana Pavlova, Lika Fab, Dzhirgala Shamadykova, Alexander Revishchin, Anna Alekseeva, Alexey Kopylov, Igor Pronin, Galina Pavlova

**Affiliations:** 1 Institute of Higher Nervous Activity and Neurophysiology, Russian Academy of Sciences, 117485 Moscow, Russia; 2 Institution N. N. Burdenko National Medical Research Center of Neurosurgery of the Ministry of Health of the Russian Federation, 125047 Moscow, Russia; 3 Avtsyn Research Institute of Human Morphology of Federal State Budgetary Scientific Institution, «Petrovsky National Research Centre of Surgery», 117418 Moscow, Russia; 4 Belozersky Research Institute of Physical Chemical Biology, Lomonosov Moscow State University, 119991 Moscow, Russia

**Keywords:** glioblastoma, EGFRvIII, aptamer, targeted therapy

## Abstract

**Background:** This study aimed to develop a superior aptamer-based therapeutic for targeted glioblastoma intervention by conducting a comparative analysis of two DNA aptamers: the original U2 sequence, selected against glioblastoma cells exhibiting high EGFRvIII expression, and its modified, shortened, and stabilized variant, Gol1. **Methods:** The effects of the investigated aptamers on primary human glioblastoma cells with graded receptor expression levels and on a rat 101/8 glioblastoma tissue model were rigorously studied. **Results:** The results demonstrated the significant superiority of the stabilized Gol1 aptamer, which exhibited exceptional binding affinity for the EGFRvIII receptor. Pronounced antiproliferative and antimigratory effects against EGFRvIII-positive human tumor cells, ultimately inducing complete cell death. Transcriptomic analysis revealed a sophisticated dual mechanism of action for Gol1: the specific activation of neuronal differentiation genes concurrent with the suppression of key alternative splicing factors. Crucially, in vivo confirmation showed highly selective accumulation of the FAM-labeled Gol1 aptamer exclusively within tumor tissue, with a maximum concentration gradient observed in the invasive border zone and a complete absence of accumulation in intact brain parenchyma. **Conclusions:** These comprehensive findings confirm that the Gol1 aptamer constitutes a highly promising and versatile platform for developing novel targeted theranostic strategies against glioblastoma, offering a precise approach for both diagnostic imaging and therapeutic intervention.

## 1. Introduction

Epidermal growth factor receptor (EGFR) and its mutant form, EGFRvIII, play a key role in the pathogenesis of glioblastoma, the most aggressive malignant brain tumor [1]. EGFR is involved in the regulation of critical cellular processes, including proliferation, differentiation, and survival of tumor cells by activating a number of signaling pathways [2]. EGFR and EGFRvIII are often overexpressed and activated in glioblastoma, resulting in dysregulated intracellular signaling and uncontrolled tumor growth [1].

Therapeutic strategies aimed at inhibiting EGFR/EGFRvIII, such as the use of monoclonal antibodies (e.g., cetuximab) or small-molecule tyrosine kinase inhibitors (gefitinib, erlotinib), demonstrated limited efficacy in the treatment of glioblastoma [3]. The main reason for the limited success of these treatments is the fact that after the initial successful treatments the signs of drug resistance begin to emerge [4]. Moreover, side effects that limit the use of antibodies and small-molecule tyrosine kinase inhibitors have been reported [5]. Therefore, the expansion of new approaches to circumnavigating these limitations is urgently needed.

Overall, understanding the role of EGFR and EGFRvIII in glioblastoma signaling pathways is important for developing new treatment strategies and improving of the existing approaches. One such approach may be the use of aptamers [6].

One of the well-established aptamers selected for EGFRvIII-positive cells is U2 [7,8]. Recently, Golovin et al. presented a truncated modified form of U2 aptamer, Gol1 aptamer. Gol1 has been shown to be more stable when compared to U2 aptamer and, at the same time, retained high specificity for the target receptors EGFR and EGFRvIII [9]. It was demonstrated that the binding affinity to the EGFR protein of the Gol1 aptamer is similar to that of U2 [10].

Previous studies have shown that the Gol1 aptamer exhibits a more pronounced antiproliferative effect than its predecessor U2. In human glioblastoma cell culture overexpressing EGFRvIII, Gol1 aptamer treatment decreased the expression of key genes involved in EGFR-mediated signaling pathways that activate the proliferation and viability of the cells [11,12]. In addition, when analyzing the changes in the transcriptome data of human glioblastoma cells after exposure to Gol1, changes in oncogenic signaling pathways, including those mediated by EGFR, were observed, indicating a high potential of the aptamer in antitumor therapy [9]. Thus, further study of the cellular and molecular mechanisms triggered by Gol1 aptamer treatment and its capabilities as an antitumor agent is required to further understand the potential of this aptamer for the development of new glioblastoma treatment strategies.

## 2. Materials and Methods

### 2.1. Cell Cultures

A431 and DF1 cell lines were obtained from the Institute of Cytology of the Russian Academy of Sciences, St. Petersburg. Primary cultures of human glioblastoma cells BU881, Bl, G01, Sus and primary culture of olfactory neuroepithelial lining cells OES-b were obtained from explants provided by the N.N. Burdenko National Medical Research Center for Neurosurgery (Moscow). This study was approved by the Ethics Committee of Burdenko Neurosurgical Institute, Russian Academy of Medical Sciences (No_12/2020). All subjects gave written informed consent in accordance with the Declaration of Helsinki. The cells were cultured in DMEM/F12 medium (Servicebio, Wuhan, Hubei, China) supplemented with 10% FBS (Biowest, Nuaille, France), 2 mM L-glutamic acid, (Paneco, Moscow, Russia), and 1% antibiotic solution (penicillin/streptomycin) (Corning, Corning, NY, USA) at 37 °C and 5% CO_2_. Cells were removed from culture dishes using the Versene solution (Paneco, Moscow, Russia) and 0.25% Trypsin solution (Paneco, Moscow, Russia).

### 2.2. Aptamers

Two previously characterized DNA aptamers, U2 and Gol1 [8,9], were used in this study. FAM-labeled variants of these aptamers (U2-FAM and Gol1-FAM) were used to assess the functional activity and binding specificity. The aptamers were synthesized by solid-phase chemical synthesis with 5′ terminal modification with fluorescein (FAM). Purification was performed by HPLC (high-performance liquid chromatography), purity was confirmed by mass spectrometry. Working solutions were prepared in the following buffer: 7.3 mM KCl, 2 mM MgCl_2_ in PBS (pH 7.4).

### 2.3. Transfection

Transfected human glioblastoma cell cultures G01/EGFRwt and G01/EGFRvIII were obtained according to the previously described protocol [11].

### 2.4. Competitive Aptamer/Immunocytochemical Staining

Cells were cultured on round coverslips in a 24-well plate with a preliminary 3 h incubation in the presence of U2-FAM and Gol1-FAM aptamers (concentration 1 μM) in complete growth medium DMEM/F12. After incubation, the cells were washed three times with phosphate-buffered saline (PBS, 5 min for each wash) and fixed with 4% paraformaldehyde for 30 min at +4 °C. For immunocytochemical analysis, fixed cells were incubated with primary anti-EGFR antibodies (clone 225) and anti-EGFRvIII (clone L8A4) at a dilution of 1:50 for 1 h at room temperature, followed by the incubation with secondary anti-mouse antibodies conjugated with Cy5 (1:50). Cell nuclei were counterstained with Hoechst 33342 (Sigma-Aldrich, Burlington, MA, USA, 1:500). The preparations were investigated on a confocal laser scanning microscope LSM-710 (Carl Zeiss, Oberkochen, Germany) using a femtosecond infrared laser with a tunable wavelength (800–1500 nm) for multiphoton excitation of fluorescence. Microscopy was performed with appropriate filters for detection of FAM, Cy5, and Hoechst 33342 signals.

### 2.5. RT-qPCR

A detailed protocol for primer selection and RT-qPCR is described in our previous work [9].

### 2.6. Immunocytochemistry

Cells were cultured on round coverslips in a 24-well plate. After standard fixation with 4% paraformaldehyde (30 min, +4 °C) and PBS washes, double immunostaining was performed. Primary anti-EGFR antibodies EGFR (clone H11) and anti-EGFRvIII (clone L8A4, 1:50) were detected using secondary antibodies conjugated to Alexa-488 (1:50). Nuclear staining with Hoechst 33342 (1:500) was performed in parallel. Visualization was performed on an LSM-710 confocal microscope (Carl Zeiss) using a femtosecond IR laser (800–1500 nm) and standard filters for Alexa-488 and Hoechst.

### 2.7. FAM-Aptamer Incubation and Immunohistochemistry

Cells were cultured on round coverslips in 24-well plates in complete DMEM/F12 growth medium at 37 °C and 5% CO_2_ until 70–80% confluent. To analyze the kinetics of aptamer accumulation, cells were incubated with U2-FAM and Gol1-FAM solutions (final concentration 1 μM) for 1.5, 3, 24, 48, and 72 h, using separate plates for each time point. Control samples contained cells incubated only in the growth medium without aptamers. After incubation, the medium was removed and washed three times with PBS for 5 min at 37 °C. Fixation was performed with 4% paraformaldehyde in PBS for 30 min at +4 °C, followed by three washes with PBS. To visualize the nuclear material, the cells were incubated with Hoechst 33342 dye (diluted 1:500 in PBS) and then washed twice with PBS solution. Visualization was performed on an LSM-710 confocal laser scanning microscope (Carl Zeiss) with settings for FAM detection (excitation 488 nm, detection 500–550 nm) and Hoechst 33342 (excitation 405 nm, detection 410–480 nm). Cells without aptamers (negative control) were used as a specificity control.

### 2.8. Determination of Fluorescence Intensity

Fluorescence intensity was determined by CellProfiler4.2.8 software (Broad Institute, Cambridge, MA, USA; available online: https://cellprofiler.org/ (accessed on 1 February 2026)). Images were converted to black and white using ColorToGray module. Cell boundaries were determined by IdentifyPrimaryObjects module. The original color images were then separated into channels using ColorToGray and the fluorescence intensity of cells in the desired channel was determined using MeasureObjectIntensity module. The average intensity per pixel of the object and the standard deviation were determined.

### 2.9. MTS Analysis

Changes in cell proliferative activity after exposure to aptamers were assessed by MTS assay. Cells were seeded at 2000 cells per well in 96-well plates (three replicates) in DMEM/F12 culture medium. Aptamers’ concentration in in vitro experiments was 10 and 20 μM. Prior to addition to the cell culture, aptamers were treated at 95 °C followed by cooling at room temperature for one hour. Incubation was performed at 37 °C with 5% CO_2_ for 72 h. After 72 h, the cells were washed and 100 μL culture medium plus 10 μL MTS reagent (Promega, Madison, WI, USA) was added per well. Cells were incubated at 37 °C with 5% CO_2_ for 2 h. Aptamers were not present in the positive control, and the cell medium was used as a blank. Optical density was measured at l = 495 nm using a CLARIOstar Plus tablet analyzer (BMG LABTECH, Ortenberg, Germany).

### 2.10. xCELLigence RTCA DP Cell Analysis

Human glioblastoma cells (BU881, Bl and G01) were cultured in DMEM/F12 at 37 °C under 5% CO_2_. The xCELLigence RTCA DP system (Agilent, Santa Clara, CA, USA) was used for proliferation analysis according to the manufacturer’s protocol.

Before the experiment, E-Plate 16 was coated with 50 μL of medium and calibrated in the analyzer. After that, 100 μL of cell suspension (7 × 10^3^ cells/well) was added to each well and left at room temperature for 30 min to rich uniform distribution of the cells. The plate was placed in the xCELLigence station and baseline impedance measurements (Cell Index) were taken every minute for the first 5 h, then every 15 min until the end of the first 24 h.

After 24 h of seeding, experimental aptamers (U2 and Gol1) were added to the wells at a final concentration of 10 μM. The control group contained cells without aptamers. Cell index measurements were continued every 15 min for 72 h, then every 30 min for another 72 h. Data were analyzed using RTCA 2.2.5 software (ACEA Biosciences). Cell growth curves were normalized to the time of aptamer addition (T = 24 h).

### 2.11. Migration Analysis

Cell migration activity after aptamer exposure was assessed using Transwell inserts with 8 μm pores (NEST, Wuxi, China). Cells were incubated in a serum-free medium for one hour, and then 20,000 cells per well in 350 µL of serum-free medium were transferred into each insert. Control wells contained 700 μL of culture medium with 10% FBS. After 20 h, non-migrated cells on the inner side of the insert were removed, and migrated cells on the outer side of the insert were fixed with 4% paraformaldehyde and stained with Hoechst 33342 (Sigma, USA). The migration activity was defined by cell count in six non-crossing visual fields. Wells without the addition of aptamers were used as controls.

### 2.12. Statistical Analysis

MARS 3.33 software was used to analyze MTS assay data. Expression levels of the target genes were measured by LightCycler^®^ 96 1.1 system software. GraphPad Prism 9 (Boston, MA, USA) was used for statistical analysis, and the data were expressed as mean ± SEM and the *p*-test was considered significant when * = *p* < 0.05, ** = *p* < 0.01, *** = *p* < 0.001 and **** = *p* < 0.0001. One-way analysis of variance (ANOVA) followed by Bonferroni’s multiple comparison test was used to assess the significance of the MTS assay and RT-PCR results. The change in mean fluorescence intensity and migration activity was assessed by two-way ANOVA followed by Bonferroni’s multiple comparison test.

### 2.13. Transcriptomic Analysis

Transcriptomic analysis was performed using the data from our previous study [9]. Here, we extended the analysis to delineate alterations in the key pathways, including neuronal differentiation, neurogenesis, maintenance of neural stem cell populations, and alternatively spliced genes. Protein–protein interaction (PPI) networks were constructed using STRING database (v12.0) 1 with a confidence threshold of ≥0.7.

### 2.14. Animal Models

Adult Wistar rats (males, 10–12 weeks old, weighing 250–300 g) were used in the experiment. The animals were kept under standard vivarium conditions at a temperature of 22 ± 2 °C, a 12 h light cycle, and with free access to water and food. All procedures were performed according to the rules of bioethics and approved by the local ethics committee. Experiments were performed according to the European Union Directive 2010/63/EU on the protection of animals used for scientific purposes. Animal care and use were in accordance with the institutional policies and guidelines. The study was approved by the Ethical Committee of the IHNA (protocol №3 of 12 May 2025). All efforts were made to minimize the number of animals used in experiments and their suffering from experimental procedures.

#### 2.14.1. Glioblastoma Transplantation 101/8

Glioblastoma 101/8 was transplanted into rats according to a previously described method [13].

#### 2.14.2. Introduction of Fluorescent Markers

##### Dose–Response Injection Protocol

To assess the distribution of fluorescent compounds in the brain tissues, rats with transplanted glioblastoma (post-operative day 14–18) were injected intravenously (into the tail vein) either:

- Sodium fluorescein in PBS at doses of 100, 600, and 6300 nmol/kg (*n* = 3 per group);

or

- Fluorescein-labeled Gol1 aptamer at doses of 50, 100, 200, 400, and 3800 nmol/kg (*n* = 3 per dose in the preliminary experiment).

The aptamers were dissolved in buffer (7.3 mM KCl, 2 mM MgCl_2_ in PBS, MP Biomedicals, Solon, OH, USA) and brought to a final volume of 1 mL to standardize the injection. All solutions were prepared immediately before administration.

##### Targeted Injection for Confocal Microscopy

On day 14 post-implantation, two rats were injected with Gol1-FAM (3.6 μmol/kg, i.v.).

#### 2.14.3. Brain Slices Preparation

##### Fluorescence Scanning Protocol

Protocol for scanning on a fluorescence scanner: 15 min after injection, animals were euthanized with an overdose of zoletil. The brain was frozen on a Peltier table (HM 525 cryostat, Thermo Fisher Scientific, Waltham, MA, USA), 50 μm sections were prepared.

##### Protocol for Confocal Microscopy

After euthanasia by isoflurane, brains were immediately vitrified, sectioned at 10 μm, fixed with 4% paraformaldehyde, and counterstained with Hoecht 33342.

#### 2.14.4. Visualization and Analysis

##### Fluorescence Scanning

Sections were scanned on a Typhoon FLA 9500 (GE Healthcare, Uppsala, Sweden): excitation 473 nm, emission 510 nm (LPB filter). Intensity was analyzed in ImageJ version 1.53k (NIH, USA) as the ratio of tumor fluorescence to normal tissue (IF).

##### Confocal Microscopy

Accumulation of Gol1-FAM was studied using a Carl Zeiss LSM-710 series laser (Oberkochen, Germany) scanning confocal microscope with a short-pulse femtosecond infrared laser with a tunable range (800–1500 nm) for multiphoton fluorescence excitation.

#### 2.14.5. Quantitative Fluorescence Analysis

Fluorescence intensity was assessed using ImageJ version 1.53k (NIH, USA). The fluorescence index (FI) was calculated as:

FI = I tumor/I normal

where

- I tumor—average tumor fluorescence intensity;
- I normal—intensity in normal brain tissue of a similar area.

#### 2.14.6. Statistical Analysis

To compare whether the means of the control (600 μM FAM) and experimental groups (50, 100, 200, 400, 3800 μM Gol1-FAM) were significantly different one-way ANOVA with Dunnett’s post hoc test was used. The choice of the method is justified by the need for multiple comparisons with a single control with normal data distribution (confirmed by the Shapiro–Wilk test, *p* > 0.05) and homogeneity of variances (Levene’s test, *p* = 0.21).

Data were presented as mean ± SD (*n* = 3 biological replicates). The analysis was performed in GraphPad Prism 9.0 (GraphPad Software, USA). The results were visualized as a bar chart with SD and significance symbols * = *p* < 0.1, ** = *p* < 0.01, *** = *p* < 0.001, **** = *p* < 0.0001.

## 3. Results

### 3.1. Determination of the Specificity of the Gol1 Aptamer to EGFR and EGFRvIII

**Figure 1:**
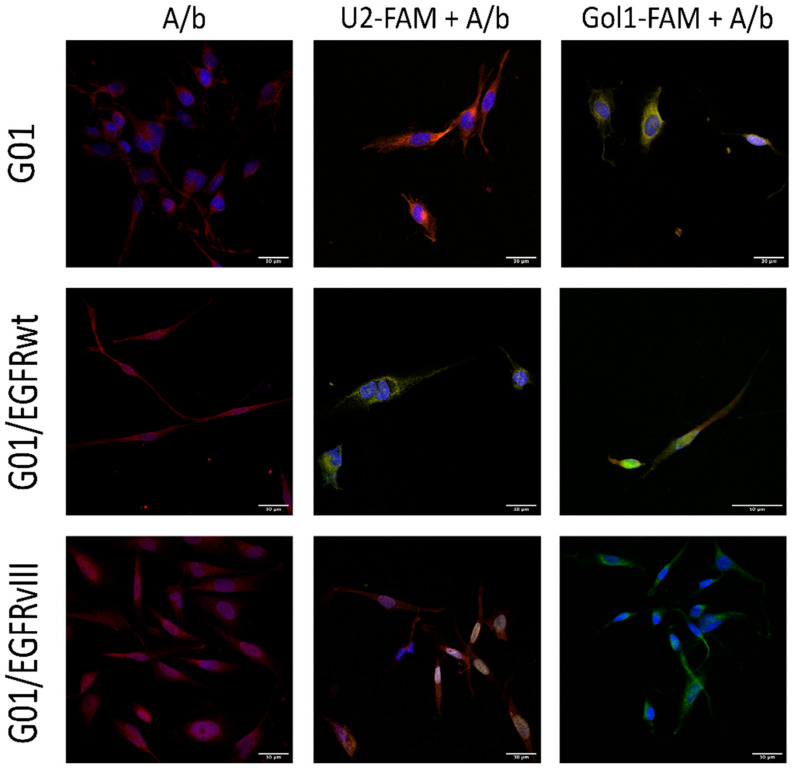
Comparative analysis of U2-FAM and Gol1-FAM aptamers (green) binding to EGFR in human glioblastoma G01, G01/EGFRwt, and G01/EGFRvIII cells using competitive apta-immunocytochemistry. Nuclear staining (blue): Hoechst 33342 (Sigma-Aldrich). Yellow-orange indicates colocalization of aptamers with EGFR/EGFRvIII antibodies (A/b). Scale bar length 30 μm.

To determine the specificity of the U2 and Gol1 aptamers for wild-type EGFR and its mutant isoform EGFRvIII, we performed a competitive apta-immunocytochemical staining assay using three cell lines: parental human glioblastoma G01 cells (derived from patient tumor tissue), as well as G01 cells stably transfected with EGFRwt (G01/EGFRwt) or EGFRvIII (G01/EGFRvIII) (Figure 1, Supplementary Figure S1, Supplementary Table S1).

First, live cells were incubated with either FAM-labeled U2 (U2-FAM) or FAM-labeled Gol1 (Gol1-FAM) aptamers for 3 h to allow binding to their targets. After fixation, the cells were stained with antibodies against EGFR and EGFRvIII. Since all other experimental conditions remained unchanged, the observed reduction in antibody binding following aptamer pre-incubation indicates that the aptamers occupy specific epitopes on the receptors, demonstrating their competitiveness for the binding sites.

Human glioblastoma G01 cells were incubated with either U2-FAM (green) or Gol1-FAM (green) aptamers. Competitive apta-immunocytochemical staining of G01/U2-FAM and Go1/Goll-FAM cells with anti-EGFR (red) antibodies revealed that when U2-FAM aptamer was used the red fluorescent signal predominated (~2 times). In contrast, Gol1-FAM treated cells show the prevalence of the green signal (~2 times). The binding of EGFR to the competing antibody is minimal in Gol1-FAM treated cells, which may indicate high competition and receptor specificity (Figure 1, Supplementary Table S1).

Based on our data, Gol1 exhibited high competition with anti-EGFR antibodies for binding to EGFR on the surface of G01 glioblastoma cells. To establish the aptamers binding specificity to wild-type and mutant receptors, we generated G01 glioblastoma cell lines overexpressing EGFR and mutant EGFRvIII. A comparative study of these cell lines was repeated by competitive apta-immunocytochemical staining using Gol1-FAM and U2-FAM aptamers incubation with the cells followed by immunostaining with anti-EGFR and anti-EGFRvIII antibodies. Apta-immunocytochemical analysis (Figure 1, Supplementary Table S1) of the transfected G01/EGFRwt cell line demonstrated that aptamer U2-FAM exhibited the same high affinity to the cells overexpressing EGFRwt as the monoclonal antibodies (Figure 1, Supplementary Table S1), while aptamer Goll-FAM showed approximately 2 times less fluorescence intensity when compared to the antibodies (Figure 1, Supplementary Table S1). The obtained data suggest that aptamer U2 has exceptional EGFRwt selectivity. While Gol1 retained the ability to specifically interact with the target receptor, its competitive binding efficiency might be inferior to that of U2.

A similar experiment was performed using a transfected G01/EGFRvIII culture. U2-FAM and Gol1-FAM aptamers were incubated with the cells followed by staining with anti-EGFRvIII antibodies. Our data showed that the Gol1 aptamer exhibits high specificity for cells overexpressing the EGFRvIII mutant (Figure 1, Supplementary Table S1). The intensity of the fluorescent signal from Gol1 is 4.2 times higher that of EGFRvIII monoclonal antibodies. Figure 1 showed that there was no overlapping of the red signal from anti-EGFRvIII antibodies and the green signal from the Gol1-FAM aptamer indicating that the Gol1 aptamer, in addition to being highly specific for EGFRvIII, is more competitive in binding to the target molecule than antibodies. In the case of the U2-FAM aptamer (Figure 1, Supplementary Table S1), an overlap of the red and green channels was observed; however, the red signal intensity from antibodies to EGFRvIII was more pronounced (approximately 1.4 times).

In summary, we showed that U2 aptamer exhibits high specificity for the wild-type EGFR, while the Gol1 aptamer is highly specific for the mutant form EGFR vIII mutant form.

### 3.2. Selection of Human Glioblastoma Cell Cultures with Various Expression of EGFRwt and EGFRvIII to Further Investigate the Properties of Gol1 Aptamer

**Figure 2:**
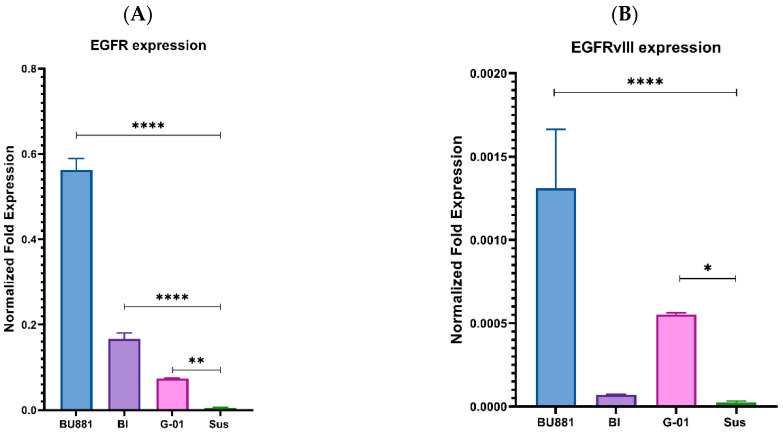
Analysis of EGFRwt (**A**) and EGFRvIII (**B**) expression levels in human glioblastoma BU881, Bl, G01 and Sus cells by qPCR. Relative expression levels were normalized to the geometric mean of three housekeeping genes (GAPDH, RPL13A, and TBP). Data are presented as mean ± SD; *n* = 3 for each group. Statistically significant differences between groups are indicated by asterisks (multiple unpaired *t*-tests, **** = *p* < 0.0001, ** = *p* < 0.01, * = *p* < 0.05).

We selected four human glioblastoma cell cultures BU881, Bl, G01 and Sus, based on the various expression levels of EGFRwt and EGFRvIII. The analysis of EGFRwt and EGFRvIII expression was performed by qPCR (Figure 2A,B). Notably, we already reported the expression levels of these receptors in BU881 and G01 cell cultures in our previous study [9].

**Figure 3:**
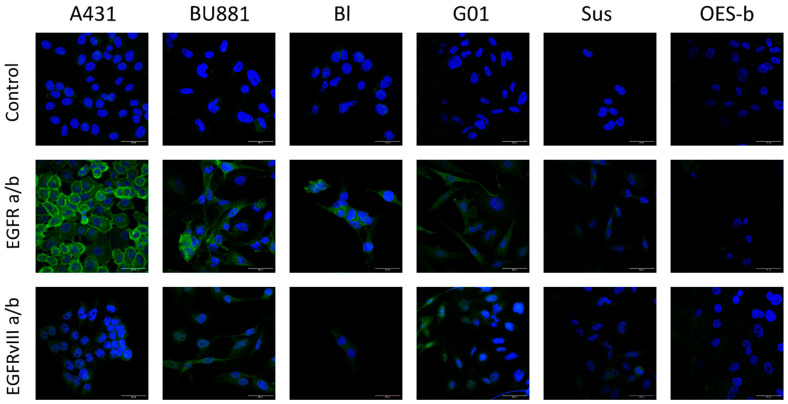
Immunocytochemical staining of human glioblastoma cell cultures BU881, Bl, G01 and Sus using anti-EGFR and anti-EGFRvIII antibodies (a/b) (green). Positive control—A431 cells; negative control—OES-b neuro-olfactory mucosa culture cells. Blue—nuclear stain Hoechst 33342 (Sigma-Aldrich). Scale bar length 50 μm.

The highest expression levels of EGFR and EGFRvIII were demonstrated in the BU881 cells while the lowest expression was observed in Sus cells. Sus cells were used as an additional negative control for EGFR and EGFRvIII receptor expression levels (Figure 2A,B). The key cell cultures for our experiments were Bl and G01 based on the differential expression of wild-type and mutant EGFRvIII receptors. The Bl culture cells exhibited higher EGFR expression than G01 cells (Figure 2A) but showed lower EGFRvIII expression (Figure 2B). This selection of cell cultures allowed us to use differential assessment of the effects mediated by the wild type and mutant receptors, which provided a more accurate analysis of their functional contribution to glioblastoma biology. Immunocytochemical staining of cells with antibodies against EGFR and EGFRvIII was performed using this panel of human glioblastoma cell cultures to assess protein abundance (Figure 3).

Our data demonstrated that the distribution of EGFR in the cells of the A431 adenocarcinoma line is predominantly membranous, whereas a predominantly diffuse distribution of the receptor in the cytoplasm was observed in all selected human glioblastoma cultures (Figure 3). In general, the receptor expression level obtained by qPCR correlated with the immunocytochemistry data, which gave us an opportunity to use this cell panel for further experiments.

### 3.3. Distribution of the Gol1 Aptamer in Human Glioblastoma Cells

**Figure 4:**
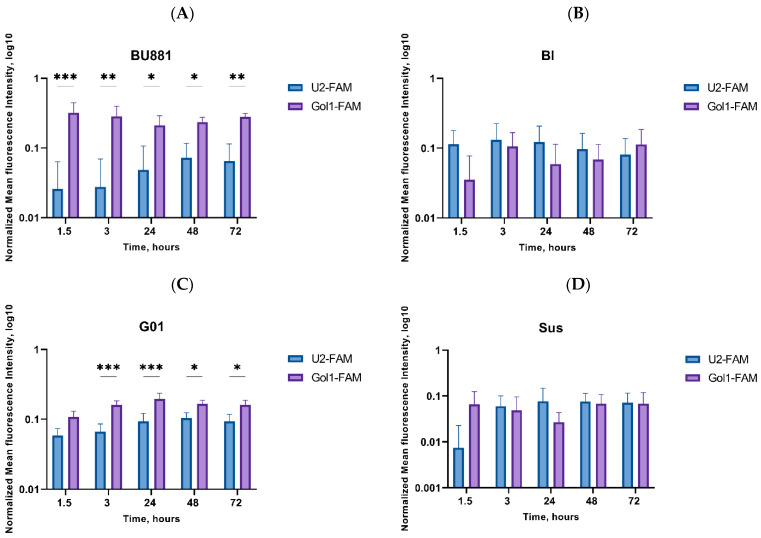
Normalized mean fluorescence intensity of U2-FAM and Gol1-FAM aptamers in human glioblastoma cells BU881 (**A**), Bl (**B**), G01 (**C**) and Sus (**D**) after 1.5, 3, 24, 48, and 72 h of cultivation. Normalization was performed using the background signal in control cells without the addition of the fluorescent FAM label. Statistically significant in fluorescence intensity (indicative of differential binding selectivity) between the U2-FAM and Gol1-FAM aptamers are indicated by asterisks (two-way ANOVA followed by Bonferroni’s multiple comparison test, * = *p* < 0.05, ** = *p* < 0.01, *** = *p* < 0.001).

To analyze the distribution of Gol1 and U2 aptamers we analyzed the fluorescence intensity of BU881, Bl, G01 and Sus after 1.5, 3, 24, 48 and 72 h of incubation with aptamers labeled with a fluorescent FAM label (Figure 4A–D, Supplementary Figure S2).

We observed significant differences in the binding of the two aptamers in human glioblastoma BU881 and G01 cultures, characterized by high EGFRvIII expression (Figure 4A,C). The Gol1 aptamer exhibited a higher affinity for these cultures than the U2 aptamer. As expected, a positive correlation was found between the level of the EGFRvIII expression level and Gol1-FAM fluorescence intensity. The high binding and accumulation of the Gol1 aptamer in these cultures was maintained throughout the observation period (from 1.5 to 72 h). In contrast, no statistically significant differences were observed between U2 and Gol1 cells that are characterized by low expression of EGFRvIII (Figure 4B,D). Our results indicated the Gol1 aptamer’s selectivity for cells with high expression of EGFRvIII, which was confirmed by a prolonged and stable binding signal over a long period of incubation with the aptamers.

### 3.4. Effect of Gol1 Aptamer on the Proliferative Potential of Human Glioblastoma Cells

#### 3.4.1. MTS-Proliferation Test

**Figure 5:**
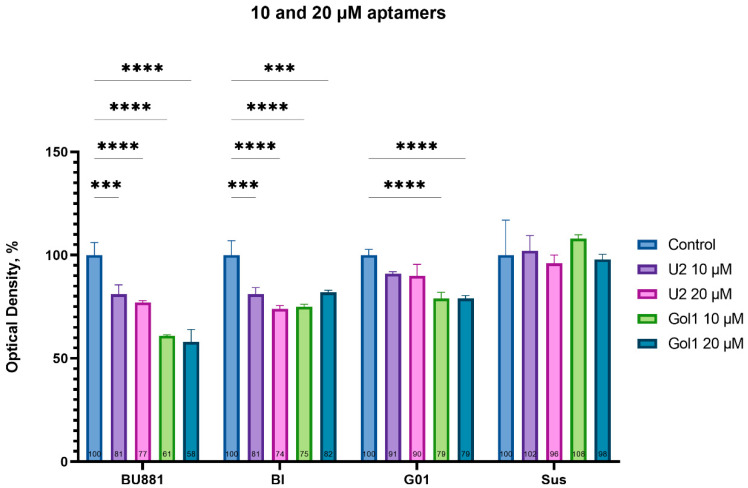
Effects of U2 and Gol1 aptamers (10–20 μM) on proliferation of human glioblastoma cells (BU881, Bl, G01, Sus) assessed by MTS assay. Data expressed as percentage of untreated control (100%). Statistical significance versus control: *** *p* < 0.001, **** *p* < 0.0001 (one-way ANOVA with Bonferroni correction).

Our previous comprehensive analysis provided us with valuable information about the binding kinetics and accumulation of U2 and Gol1 aptamers in four human glioblastoma cell cultures. To further assess U2 and Gol1 aptamers effect on the proliferative activity of these cell lines we performed a standard MTS test after 72 h of cell incubation in the presence of one of the aptamers at concentrations of 10 and 20 μM (Figure 5).

The results of this experiment demonstrated significant differences in the antiproliferative activity of the U2 and Gol1 aptamers. In primary human glioblastoma BU881 cells, a pronounced inhibitory effect of the Gol1 aptamer (∼40% decrease in proliferation) was observed at both experimental concentrations, significantly exceeding the activity of the original U2 aptamer. Upon treatment with U2 aptamers, G01 cells have not shown a statistically significant changes in proliferation rate, while the modified Gol1 aptamer caused a reliable decrease in proliferative activity by 21%. In the Bl culture, both compounds demonstrated comparable efficacy, whereas in the Sus cells, characterized by minimal expression of the target receptors, no significant antiproliferative effect was observed.

**Figure 6:**
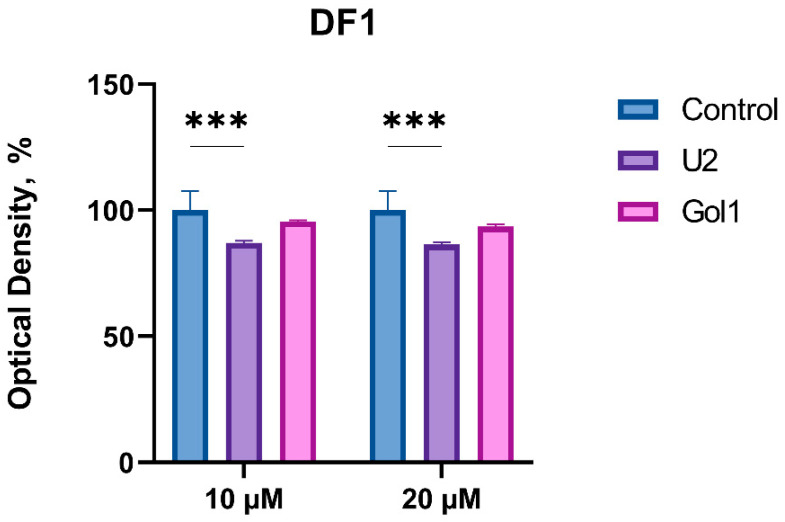
Effects of U2 and Gol1 aptamers (10–20 μM) on proliferation of DF1 (human fibroblasts) cells assessed by MTS assay. Data expressed as percentage of untreated control (100%). Statistical significance versus control: *** *p* < 0.001 (one-way ANOVA with Bonferroni correction).

Our data demonstrate that there is a correlation between the level of EGFRvIII expression and cell sensitivity to the Gol1 aptamer, which confirms its selectivity for this receptor isoform. However, an ideal therapeutic molecule should effectively inhibit tumor cell division while exerting minimal toxic effects on normal cells. Therefore, to assess the cytotoxic effect of U2 and Gol1 aptamers on normal tissue cells, their effect on the metabolic activity of DF1 fibroblast cell line was analyzed (Figure 6). We previously showed that neither U2 nor Gol1 aptamer has an antiproliferative effect on primary olfactory neuroepithelial cells OES-b at a concentration of 20 μM [9].

Analysis of the proliferative activity of DF1 cells using the MTS test (Figure 6) showed that the Gol1 aptamer did not have a statistically significant effect on the metabolic activity of this cell line, while the U2 aptamer exhibited a moderate antiproliferative effect (the inhibition level was ~14%).

These results indicate that the Gol1 aptamer possesses a higher selectivity for tumor cells compared to the U2 aptamer and exhibits a more favorable safety profile for normal cells.

These data confirm the promise of further study of the Gol1 aptamer as a potential therapeutic agent for glioblastoma targeted therapy.

#### 3.4.2. Real-Time Cell Analyzer Analysis

**Figure 7:**
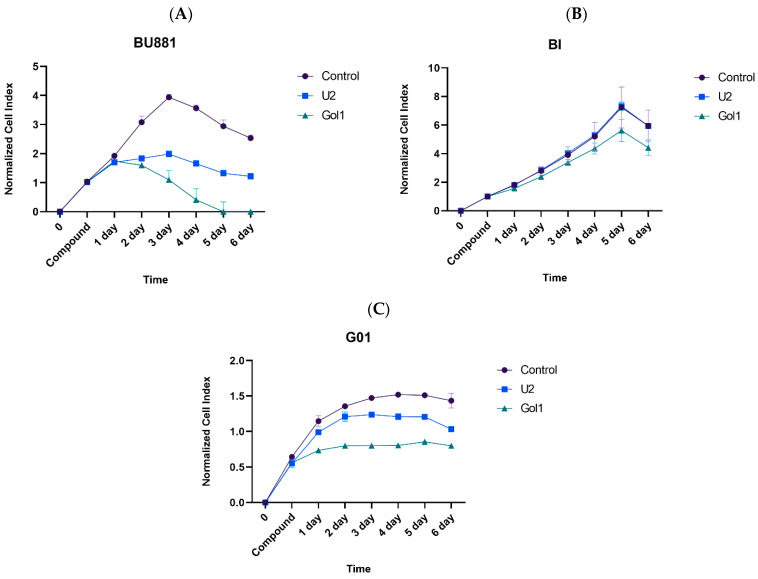
Evaluation of U2 and Gol1 aptamer effects on human glioblastoma. Cellular index of BU881 (**A**), Bl (**B**) and G01 (**C**) human glioblastoma cells after incubation with either U2 or Gol1 aptamers (10 μM) for 6 days.

MTS assay measures the metabolic activity of cells (reduction in tetrazolium salt), which only indirectly reflects their proliferation. The results may be distorted by changes in cell metabolism unrelated to proliferation. In addition, MTS assay provides a single measurement at a fixed point in time, precluding the monitoring of the dynamics of the cellular response. To overcome these methodological limitations, we studied the effect of U2 and Gol1 aptamers on the proliferation of human glioblastoma cell cultures using the xCELLigence system, which enables the real-time analysis with multiple time-course measurements (Figure 7A–C).

The results of the real-time analysis confirm our MTS assay data, demonstrating a pronounced antiproliferative effect of both aptamers in human glioblastoma BU881 cells (Figure 7A). However, comparative analysis revealed significant differences in their biological activity; U2 exhibited a moderate inhibitory effect, which is consistent with previous observations. Gol1 demonstrated significantly more pronounced efficacy leading to a progressive decrease in proliferative activity and a complete loss of cell viability by the 5th day of incubation.

The analysis of cell proliferation dynamics in human glioblastoma Bl cell culture revealed no significant changes in the cell index level during the first 72 h of incubation with either U2 or Gol1 aptamers. However, prolonged exposure revealed differential effects: while U2 maintained proliferation rates comparable to untreated controls, the Gol1 aptamer exhibited a clear inhibitory effect on cell proliferation between days 4–5 of treatment (Figure 7B). This delayed response pattern suggests that the antiproliferative activity of Gol1 requires extended exposure time to manifest in this particular cell line, contrasting with the lack of effect observed for the U2 aptamer throughout the experimental period.

The human glioblastoma G01 cell culture demonstrated comparable but less pronounced effects than those observed in BU881 cells; this attenuated response likely reflects the lower expression levels of target receptors in this particular cell line (Figure 7C). Both U2 and Gol1 aptamers significantly reduced the cellular index compared to untreated controls, with the Gol1 aptamer showing consistently stronger antiproliferative effects across all tested conditions. The differential potency between the two aptamers suggests that Gol1 may have enhanced binding affinity or more efficient downstream signaling modulation despite the reduced receptor availability in G01 cells. These findings align with our previous observations of Gol1’s superior therapeutic potential while highlighting the importance of target receptor density in determining treatment efficacy.

### 3.5. Evaluation of the Effect of Gol1 Aptamer on the Migration of Human Glioblastoma Cells

**Figure 8:**
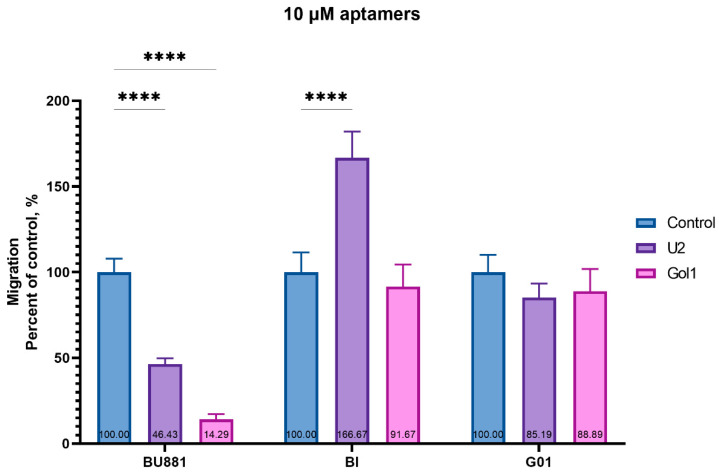
Evaluation of U2 and Gol1 aptamer effects on human glioblastoma. Migration of BU881, Bl, and G01 glioblastoma cells after 72 h treatment with U2 and Gol1 aptamers in transwell assays. Data normalized to untreated controls (100%). **** *p* < 0.0001 vs. control (two-way ANOVA + Bonferroni).

Next, we evaluated the effect of the U2 and Gol1 aptamers on the migration activity of human glioblastoma cell cultures BU881, Bl, and G01 (Figure 8).

Our data (Figure 8) demonstrated that both aptamers Gol1 and U2 significantly suppressed the migration of BU881 culture cells (by 85.5% and 53.5%, respectively). Gol1 had no statistically significant effect on the migration in Bl and G01 culture cells; however, U2 increased the migration of Bl culture cells by almost 67%, despite the decrease in proliferation observed in Bl cells after treatment (Figure 5).

### 3.6. Transcriptome Data Analysis of Human Glioblastoma Cells After Treatment with Gol1 Aptamer

Our previous work [9] demonstrated that treatment with the Gol1 aptamer caused profound changes at the transcriptional level in human glioblastoma G01 cells.

Our data revealed that the aptamer induced the expression of genes associated with neurogenesis and cellular differentiation and reduced the expression of genes mediating key nuclear processes. Significant changes in the transcription of genes of key pro-oncogenic signaling pathways mediated by EGFR were also observed.

In addition, increased expression of apoptotic genes was observed. One of the most pronounced up-regulated genes was found to be the gene encoding thioredoxin-interacting protein (TXNIP).

Moreover, aptamer treatment caused an increase in gene expression responsible for neuronal cellular differentiation. Supplementary Figure S3A shows the network of protein–protein interactions involved in the process of neuronal differentiation.

In addition to stimulating neuronal differentiation, the aptamer also promoted neurogenesis in the experimental sample compared to the control. The scheme of protein–protein interactions of overexpressed genes involved in neurogenesis is shown in Supplementary Figure S3B.

Interestingly, in addition to enhancing neurogenesis, Gol1 aptamer also affected the maintenance of key components of the normal neural stem cell population (Supplementary Figure S3C).

**Figure 9:**
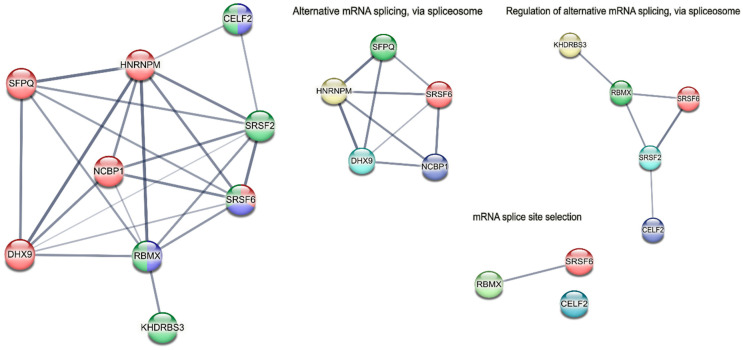
PPI network of alternative splicing-associated genes down-regulated in human glioblastoma G01 cells as a result of Gol1 aptamer treatment. PPI—protein–protein interaction; STRING—a search tool for extracting interacting genes. Network Statistics: number of nodes: 9; number of edges: 23; average node degree: 5.11; avg. local clustering coefficient: 0.885; expected number of edges: 2; PPI enrichment *p*-value: <1.0 × 10^−16^.

Finally, we observed a decrease in the expression of genes associated with alternative splicing (Figure 9).

### 3.7. Visualization of the Gol1-FAM Aptamer in Animal Models with Implanted 101/8 Glioblastoma Tissue

To evaluate the fluorescence intensity of the Gol1-FAM aptamer in animal models with implanted 101/8 glioblastoma tissue, the accumulation of pure sodium fluorescein in the tumor area of 101/8 rats was measured (Supplementary Figure S3D–F).

This experiment demonstrated dose-dependent tumor visualization. At a dose of 100 nmol/kg, tumor contours were virtually indistinguishable, with increased fluorescence intensity observed in the tumor center. At a dose of 600 nmol/kg, tumor borders were clearly delineated, and sodium fluorescein distribution was uniform. At a dose of 6.3 μmol/kg, tumor visualization was most distinct. Quantitative intensity values are provided in Supplementary Table S2.

**Figure 10:**
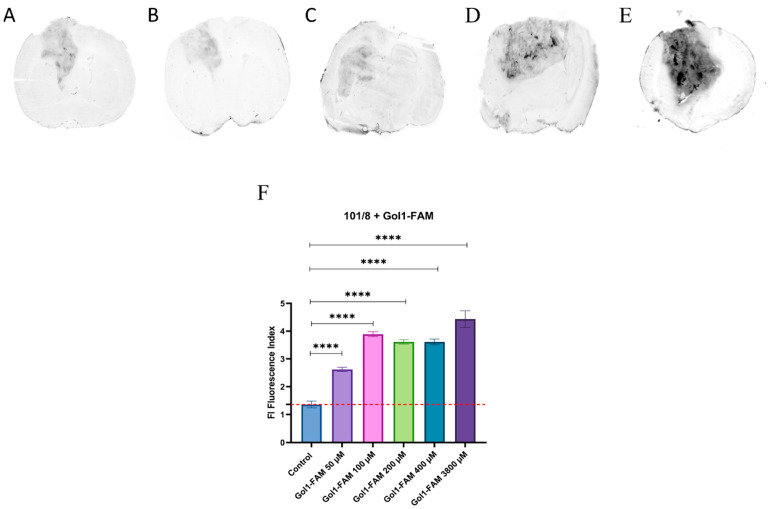
In vivo biodistribution of Gol1 aptamer after systemic administration. Fluorescence scanning of the cryosections of the rat brains (*n* = 3) implanted with glioblastoma 101/8 after administration of the Gol1 aptamer at a dose of 50 (**A**), 100 (**B**), 200 (**C**), 400 (**D**) nmol/kg and 3800 nmol/kg (**E**) (section thickness 50 μm). Dose-dependent accumulation of aptamer-FAM compared to control (600 μM FAM) in tumor zone 101/8 (**F**). One-way analysis of variance (ANOVA) with Dunnett’s post hoc test was used to compare all experimental groups with the control. Data are presented as mean ± SD (*n* = 3), **** = *p* < 0.0001.

To assess the efficiency and target localization of the Gol1 aptamer labeled with a fluorescent FAM tag, animals with implanted glioblastoma 101/8 were systemically administered solutions of the Gol1-FAM aptamer at doses of 50, 100, 200, 400, and 3800 nmol/kg (three animals per point) on days 14–18 after tumor implantation. In all cases, a significant increase in the fluorescence level was observed compared to sodium fluorescein at a concentration of 600 nmol/kg (Figure 10A–F, Supplementary Table S3).

Given the small sample size (*n* = 3), we consider these results preliminary. However, the observed specific accumulation of Gol1-FAM in the tumor area of 101/8 rats provided a first insight in the accumulation of the FAM-labeled aptamer in the implanted glioblastoma in the rat brains.

### 3.8. Analysis of the Accumulation of Fluorescent Aptamer Gol1 in Glioblastoma 101/8 Cells upon Systemic Administration

**Figure 11:**
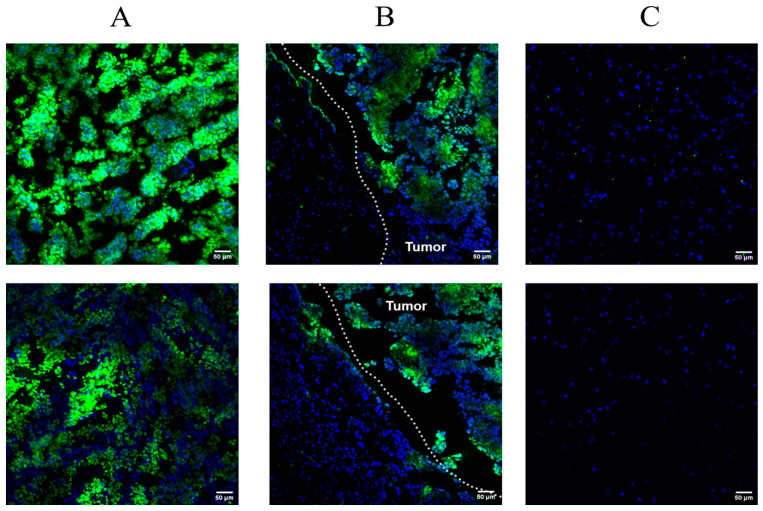
In vivo biodistribution of Gol1 aptamer after systemic administration. Analysis of the distribution of Gol1-FAM aptamer (green) in the 101/8 tumor (**A**) 15 min after intravenous administration at the border of the 101/8 tumor and intact brain tissue (**B**) and around normal brain tissue (**C**). Blue: staining of nuclei with bisbenzimide Hoechst 33342 (Sigma). Confocal laser scanning microscopy. Scale bar: 50 μm. Magnification: ×200. The dotted line highlights the border between tumor and normal brain tissue.

For a more detailed assessment of the specificity and mechanism of aptamer accumulation in tumor tissue, we performed an analysis of Gol1-FAM accumulation in 101/8 rat tumors (*n* = 2) 15 min after systemic administration using confocal laser scanning microscopy (Figure 11A–C).

Our experiment revealed selective accumulation of Gol1-FAM in 101/8 tumor tissues with pronounced heterogeneity of distribution, which may be due to the limited diffusion time of the drug (Figure 11A). Of particular interest is the detection of a fluorescent signal in individual cellular elements at the border of tumor tissue with normal tissue and outside the main tumor mass, which may indicate a specific interaction with invasive tumor cells, that play a key role in the process of tumor dissemination (Figure 11B). At the same time, we did not record a reliable accumulation of the fluorescent label in intact areas of brain tissue, which confirms the high selectivity of the aptamer for the tumor cells (Figure 11C). The data indicate a high affinity of the Gol1 aptamer for glioblastoma 101/8 cells and its potential for further study as a targeted agent for the diagnosis and therapy of glioblastoma. Of particular interest is the ability of the Gol1 aptamer to interact specifically with the invasive tumor cell population.

## 4. Discussion

Our study demonstrates that the Gol1 aptamer, which we previously developed, exhibits high specificity for EGFRvIII-rich human glioblastoma cells (Figure 1). EGFRvIII-expressing tumor cells exhibit the properties of tumor stem cells and are positively associated with tumor aggressiveness and drug resistance [14,15]. We showed that treatment of human glioblastoma BU881 cells, which are characterized by high expression of the wild-type EGFR and its mutant form EGFRvIII, with Gol1 aptamer caused not only the inhibition of proliferation but also death of the cell population. This indicates that Gol1 has the potential to exert a cytotoxic effect on the most aggressive component of the tumor (Figure 5 and Figure 6). These data confirm our initial assumption that Gol1 possesses a higher therapeutic efficacy than its original form U2. We also demonstrated that biological activity of aptamers depends on the expression level of their molecular targets within tumor cells. The data are consistent with our hypothesis that structural optimization of the parent aptamer U2 allows us not only to preserve but also to enhance its functional activity against EGFR/EGFRvIII-positive tumors [9]. When developing antitumor drugs, their ability to suppress proliferation is traditionally assessed, but, in our opinion, it is equally important to study the effect of the drugs on cell migration. These processes are interconnected through the “go or grow” phenomenon [16,17] in which under stress conditions (hypoxia, therapy) cells can switch from division to migration [18]. A comprehensive analysis of both processes is extremely important when investigating glioblastoma, an aggressive tumor whose invasiveness determines recurrence [19].

On the third day of treatment, Gol1 aptamer reduced the migration of human glioblastoma BU881 cells by 85.5% (Figure 8), while concurrently decreasing the proliferation of these cells (Figure 5 and Figure 7A). Notably, Bl cells, despite the inhibitory effect of the U2 aptamer on proliferation (Figure 5), exhibited stimulation of migration activity (Figure 8). This may indicate a possible activation of cell movement mechanisms in response to the suppression of proliferation, which is consistent with the “go or grow” concept. The obtained result emphasizes the importance of a comprehensive assessment of both proliferative and migration activity when testing new therapeutic agents. Transcriptome analysis of human glioblastoma G01 cells revealed significant changes in gene expression after Gol1 aptamer treatment. We observed the activation of genes associated with apoptosis, neurogenesis and differentiation of neuronal cells (Figure 9, Supplementary Figure S3A–C). Of particular interest is the induction of the TXNIP gene, a known tumor suppressor gene [20,21,22], whose overexpression correlates with increased apoptosis [23,24]. Reduced TXNIP expression is known to be a marker of many malignant neoplasms and is associated with an unfavorable prognosis [25,26,27,28,29].

Low cell differentiation is known to be one of the key features of malignant neoplasms. One of the current strategies in cancer therapy is differentiation therapy [30,31]. Gol1 aptamer has demonstrated the ability to induce neuronal differentiation, which is especially significant in the context of antitumor therapy. We found the activation of key differentiation genes including NOTCH3, PTN and NGF. NOTCH3 plays a critical role in neural crest cell differentiation [32], while PTN and NGF are involved in neurogenesis and maintenance of the neuronal phenotype [33,34,35]. In parallel, we observed the stimulation of neural stem cell genes (HOOK3, AspM, Prox1) (Supplementary Figure S3C). HOOK3 expression is one of the genes whose expression is known to be reduced in high-grade gliomas [36], while AspM and Prox1 play key roles in neurogenesis [37,38,39,40]. The reduced expression of genes associated with alternative splicing (Figure 9) is interesting as dysregulation of alternative splicing is increasingly observed in cancer-related pathways [41,42,43]. CELF2 (CUGBP Elav-Like Family Member 2) plays an important role in regulating alternative splicing of exon 5 of cardiac troponin T (cTNT) [44]. CELF2 protein is an effective epigenetic regulator of genes in glioma stem cells (GSC). CELF2 forms an H3K9me3-repressive landscape in the SOX3 gene, thereby promoting the proliferative phenotype of tumor cells. CELF2 has been found to be a major tumor vulnerability point, as its repression is sufficient to convert aggressive glioblastoma tumor cells into tumor-incapable cells in vivo [45]. The RNA-binding protein hnRNPM promotes breast cancer metastasis by controlling alternative splicing that occurs during epithelial–mesenchymal transition (EMT). hnRNPM is associated with aggressive breast cancer and correlates with elevated CD44 levels in patient samples [46]. KHDRBS3 (KH RNA Binding Domain Containing, Signal Transduction Associated 3) is an RNA-binding protein that plays a role in regulating alternative splicing and influencing mRNA splice site choice. KHDRBS3 was successfully identified as a gene up-regulated in cancer stem cell populations, and KHDRBS3 protein was identified as a key mediator of cancer stem cell maintenance and temozolomide resistance in glioblastoma cell lines [47]. SRSF6 (serine and arginine rich splicing factor 6) is a potential promoter of oncogenetic splicing [48]. SRSF2 (serine and arginine rich splicing factor 2) is highly expressed in colorectal cancer cells compared to normal cells, putatively participating in tumorigenesis by regulating alternative splicing [49]. Since EGFRvIII is a truncated splicing variant of EGFR [50], one of the possible mechanisms by which EGFRvIII expressing human glioblastoma cells are suppressed by the Gol1 aptamer might be the inhibition of alternative splicing.

To study the Gol1 aptamer’s efficacy and targetability upon systemic administration, a 101/8 rat glioblastoma model was chosen. The choice of this model is particularly relevant, as it closely recapitulates the biological and molecular features of human glioblastomas, thereby providing valuable data for the potential clinical translation of these aptamers [13]. By systemically administering different doses of aptamer on days 14–18 after tumor implantation we observed a significant increase in the fluorescence level compared to the control substance, sodium fluorescein (FAM) (Figure 10). These data confirm the ability of the Gol1 aptamer to selectively accumulate in the tumor parenchyma.

An interesting observation was the presence of a fluorescent signal in individual cellular elements at the border of tumor and normal tissue, as well as outside the main massive tumor (Figure 11B). This may indicate a specific interaction of the aptamer with invasive tumor cells, which play a critical role in the process of tumor dissemination and progression. The high selectivity of the Gol1 aptamer for tumor cells, along with the absence of fluorescent label accumulation in intact areas of brain tifssue, emphasizes its potential as a diagnostic and therapeutic agent. This opens new horizons for the development of targeted therapy aimed at specific molecular targets, which is crucial in the fight against aggressive forms of glioblastoma, where traditional approaches are often ineffective.

Thus, the results of our study not only confirm the efficacy of the Gol1 aptamer but also highlight its potential as a key component in innovative strategies for the diagnosis and treatment of human glioblastoma.

## 5. Conclusions

In this study, we demonstrated that the structurally optimized Gol1 aptamer exerts a potent and multifaceted antitumor effect against EGFRvIII-positive glioblastoma. Gol1 selectively binds to EGFRvIII-rich tumor cells, effectively inhibiting proliferation and inducing cell death, thereby confirming its enhanced therapeutic potential over the parental U2 aptamer. Beyond its cytotoxic effects, Gol1 suppressed tumor cell migration by 85.5%, counteracting the “go or grow” phenomenon and addressing a critical factor in glioblastoma invasiveness and recurrence.

Transcriptomic analysis further revealed that Gol1 activates key genes associated with apoptosis, neurogenesis, and neuronal differentiation, including *TXNIP*, *NOTCH3*, *PTN*, and *NGF*. This positions Gol1 as a potential agent for differentiation therapy, offering a strategy to reprogram the aggressive phenotype of tumor cells. Additionally, we observed downregulation of genes involved in alternative splicing, such as *CELF2*, *hnRNPM*, *KHDRBS3*, and *SRSF6*, suggesting a novel mechanism by which Gol1 may counteract the oncogenic splicing programs driven by EGFRvIII.

In vivo studies using a rat glioblastoma model confirmed the ability of systemically administered Gol1 to selectively accumulate in the tumor parenchyma and specifically label invasive tumor cells at the margins. This high selectivity, combined with the absence of accumulation in healthy brain tissue, underscores its potential as both a diagnostic and a therapeutic agent.

In conclusion, the Gol1 aptamer combines targeted cytotoxicity, migration blockade, and phenotypic reprogramming. Its high specificity and ability to penetrate the tumor microenvironment position it as a promising candidate for precision theranostics in aggressive glioblastoma, offering a dual-strategy approach to combat tumor recurrence and invasiveness.

## Data Availability

The original contributions presented in the study are included in the article/Supplementary Material, further inquiries can be directed to the corresponding authors.

## Supplementary Materials

The following supporting information can be downloaded at: https://www.mdpi.com/article/10.3390/pharmaceutics18030299/s1, Figure S1: Scheme of competitive aptamer-immunocytochemical staining in human glioblastoma cells; Figure S2: Apta-Cytochemical staining of human glioblastoma BU881, Bl, G01 and Sus cells using aptamers U2 and Gol1 with a fluorescent FAM label (green); Figure S3: PPI network of genes associated with neuronal cell differentiation identified using STRING, neurogenesis and maintenance of neural stem cell population obtained using STRING; Table S1: Competition of the fluorescent signals from antibodies; Table S2: Quantitative assessment of tumor fluorescence intensity after administration of various doses of sodium fluorescein, expressed as the ratio of fluorescence intensity in the tumor (average value) to healthy brain tissue, Table S3: Quantitative assessment of tumor fluorescence intensity after administration of different doses of Gol1-FAM, expressed as the ratio of fluorescence intensity in the tumor (average value) to healthy brain tissue.
