# Supplementary material for: New Player in the Field of Glioblastoma Therapy: EGFRvIII-Specific Gol1 Aptamer Shows a Great Therapeutic Potential
Source: Pharmaceutics. 2026 Feb 27;18(3):299. doi: 10.3390/pharmaceutics18030299 (PMC13028680; doi:10.3390/pharmaceutics18030299)
Supplement: Supplementary file 1 [file pharmaceutics-18-00299-s001.zip › pharmaceutics-4160773-supplementary.pdf]

SUPPLEMENTARY MATERIALS

New Player in the Field of Glioblastoma Therapy: EG-FRvIII-Specific Gol1 Aptamer Shows a Great Therapeutic Po-tential

Dzarieva et. al

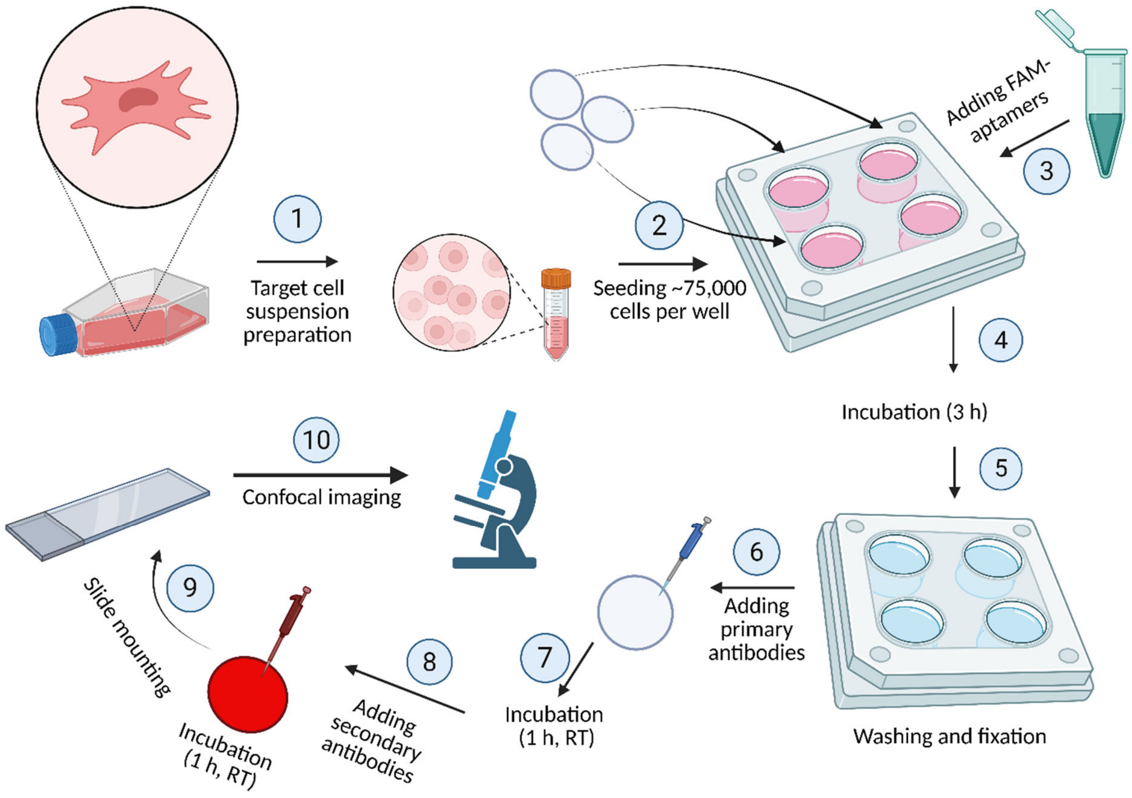

Supplementary Figure S1 –Scheme of competitive aptamer-immunocytochemical staining in human glioblastoma cells. Live cells were first incubated for 3 hours with fluorescently-labeled aptamers, followed by fixation and standard immunocytochemical staining with antibodies.

Created in BioRender. Pavlova, G. (2025) https://BioRender.com/9mhkouh

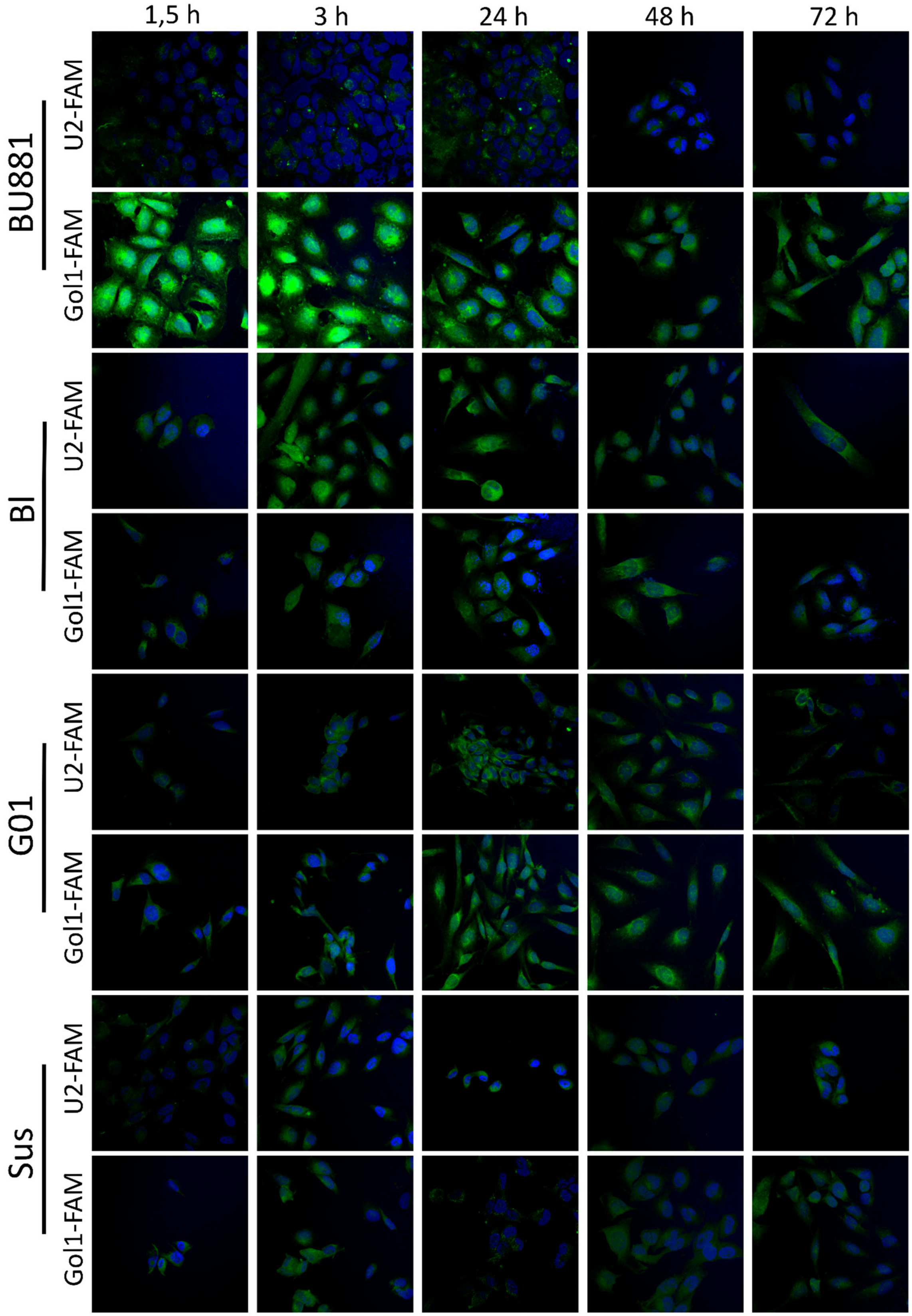
1,5 h
3 h
24 h
48 h
72 h
BU881
U2-FAM
Gol1-FAM
BI
U2-FAM
Gol1-FAM
G01
U2-FAM
Gol1-FAM
Sus
U2-FAM
Gol1-FAM

Supplementary Figure S2 - Apta-Cytochemical staining of human glioblastoma BU881, Bl, G01 and Sus cells using aptamers U2 and Gol1 with a fluorescent FAM label (green). Nuclei staining (blue) were labelled with bisbenzimide ("Hoechst 33342", "Sigma").

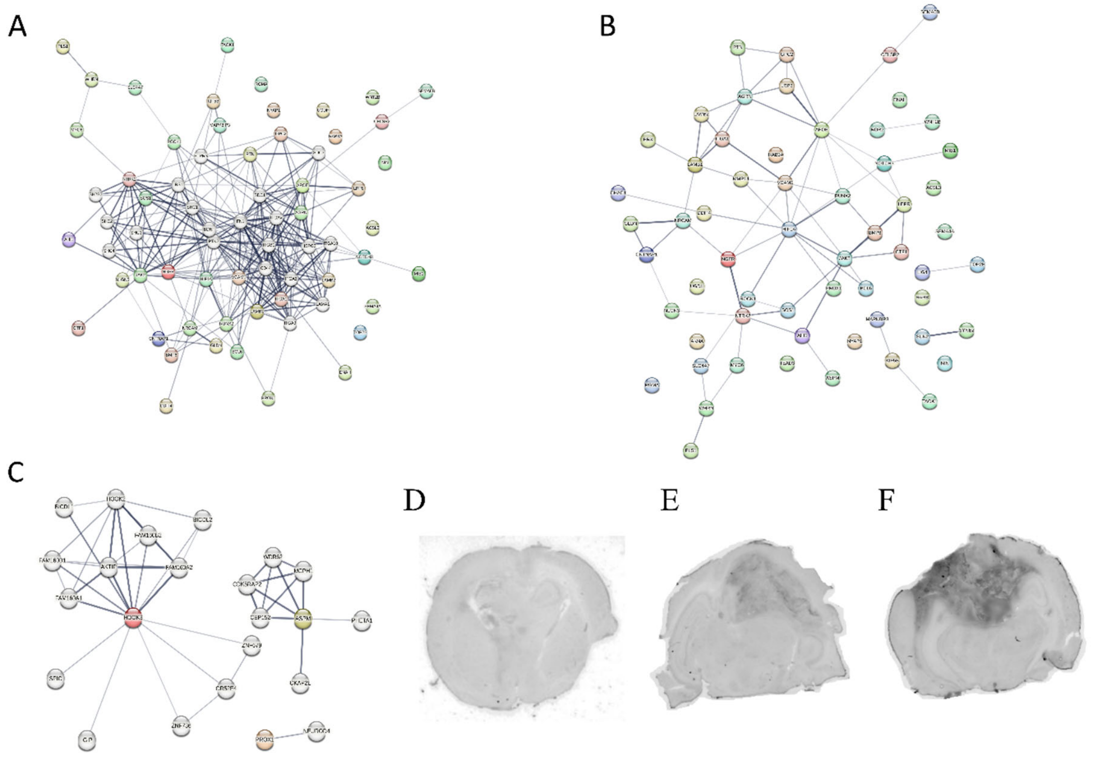

Supplementary Figure S3 – PPI network of genes associated with neuronal cell differentiation identified using STRING (**A**), neurogenesis (**B**) and maintenance of neural stem cell population (**C**) obtained using STRING. Proteins whose genes are overexpressed in the experimental sample are highlighted in color. PPI – protein-protein interaction; STRING – search tool for extracting interacting genes.

Fluorescence scanning of rat brain cryosections with implanted glioblastoma 101/8 after the introduction of sodium fluorescein at a dose of 100 (**D**), 600 (**E**) and 6300 nmol/kg (**F**), (section thickness 50 μm).

Supplementary Table S1 - Competition of the fluorescent signals from antibodies (red signal) and aptamers (green signal)

		**A/b (red) MEAN ± St. dev**	**Aptamer (green) MEAN ± St. dev**
**G01**	EGFR A/b only	0,154 ± 0,006	-
	U2-FAM+A/b	0,099 ± 0,002	0,051 ± 0,0003
	Gol1-FAM+A/b	0,056 ± 0,001	0,116 ± 0,002
**G01/EGFRwt**	EGFRwt A/b only	0,198 ± 0,004	-
	U2-FAM+A/b	0,176 ± 0,003	0,182 ± 0,002
	Gol1-FAM+A/b	0,122 ± 0,001	0,058 ± 0,0005
**G01/EGFRvIII**	EGFRvIII A/b only	0,244 ± 0,005	-
	U2-FAM+A/b	0,082 ± 0,001	0,059 ± 0,0004
	Gol1-FAM+A/b	0,038 ± 0,001	0,161 ± 0,003

Supplementary Table S2 - Quantitative assessment of tumor fluorescence intensity after administration of various doses of sodium fluorescein, expressed as the ratio of fluorescence intensity in the tumor (average value) to healthy brain tissue.

Dosage of sodium fluorescein, nmol/kg	100	600	6300
Fluorescence Index (MEAN ± St.dev.)	1,17±0,1	1,36±0,12	1,5±0,3

Supplementary Table S3 - Quantitative assessment of tumor fluorescence intensity after administration of different doses of Gol1-FAM, expressed as the ratio of fluorescence intensity in the tumor (average value) to healthy brain tissue.

Dosage of Gol1-FAM, nmol/kg	50	100	200	400	3800
Fluorescence Index (MEAN ± St.dev.)	2,62±0,08	3,89±0,09	3,61±0,08	3,61±0,1	4,43±0,3
